# Dynamics of Spirochetemia and Early PCR Detection of *Borrelia miyamotoi*

**DOI:** 10.3201/eid2405.170829

**Published:** 2018-05

**Authors:** Lyudmila Karan, Marat Makenov, Nadezhda Kolyasnikova, Olga Stukolova, Marina Toporkova, Olga Olenkova

**Affiliations:** Central Research Institute of Epidemiology, Moscow, Russia (L. Karan, M. Makenov, N. Kolyasnikova, O. Stukolova);; Medical Association Novaya Bolnitsa, Yekaterinburg, Russia (M. Toporkova);; Clinical Diagnostic Center, Yekaterinburg (O. Olenkova)

**Keywords:** dynamics, spirochetemia, PCR, detection, diagnosis, Borrelia miyamotoi, Borrelia burgdorferi, bacteria, borreliosis, Lyme disease, erythema migrans, relapsing fever, tickborne diseases, vector-borne infections, zoonoses, Russia

## Abstract

We investigated whether *Borrelia miyamotoi* disease can be detected in its early stage by using PCR for borrelial 16S rRNA, which molecule (DNA or RNA) is the best choice for this test, and whether spirochetes are present in blood during the acute phase of *B. miyamotoi* disease. A total of 473 patients with a suspected tickborne infection in Yekaterinburg, Russia, in 2009, 2010, and 2015 were enrolled in this study. Blood samples were analyzed by using quantitative PCR or ELISA, and a diagnosis of borreliosis was confirmed for 310 patients. For patients with erythema migrans, 5 (3%) of 167 were positive for *B. miyamotoi* by PCR; for patients without erythema migrans, 65 (45%) of 143 were positive for *B. miyamotoi* by PCR. The median concentration for RNA was 3.8 times that for DNA. Median time for detection of *B. miyamotoi* in blood was 4 days.

*Borrelia miyamotoi* is a relapsing fever spirochete detected in *Ixodes persulcatus* hard-bodied ticks by Fukunaga et al. in Japan in 1995 (*1*). Vectors of *B. miyamotoi* include *I. scapularis* and *I. pacificus* ticks in the United States, *I. ricinus* ticks in Europe, and *I. persulcatus* and *I. ricinus* ticks in Russia (*2**,**3*). Barbour-Stoenner-Kelly II medium has been used to isolate *B. miyamotoi* strain HT31 from *I. persulcatus* ticks and strain FR64b from *Apodemus argenteus* small Japanese field mice in Asia (*1*) and strain LB-2001 from *I. scapularis* ticks in North America (*4*). Furthermore, borreliosis caused by *B. miyamotoi* has been confirmed in Russia (*3*), North America (*5**–**8*), Europe (*9*), and Japan (*10*).

The diagnosis of Lyme disease (borreliosis) is based primarily on identification of an erythema migrans rash and positive results for serologic laboratory tests (*11*). However, PCR amplification of *B. burgdorferi* sensu lato DNA is not sensitive enough for routine diagnosis (*11**,**12*).

*B. miyamotoi* infection usually manifests as an influenza-like disease causing high fever, headache, and myalgia but, in most instances, without erythema migrans (*3*). *B. miyamotoi* produces glycerophosphodiester phosphodiesterase (glpQ), which is absent in *B. burgdorferi* sensu lato and is therefore useful for serologic discrimination between relapsing fever and Lyme borreliosis. ELISAs and confirmatory Western blot assays of human serum samples have been used to detect antibodies against *B. miyamotoi* in the Netherlands (*13*) and the United States (*14*). Wagemakers et al. (*15*) detected antibodies against variable small protein 1 and variable large proteins (Vlp15, Vlp16, and Vlp18) in blood samples of patients with *B. miyamotoi* disease. PCR and thin and thick blood smears of peripheral blood stained with Wright stain or Giemsa were used to detect infection in patients suspected of having relapsing fever (*16*).

Unlike DNA, which is usually present as a single copy per cell, or mRNAs, which constitute the minor population of total cellular RNA, each bacterium contains hundreds to thousands of 16S rRNA molecules during the growth phase. Therefore, we hypothesized that an assay based on PCR amplification of cDNA molecules, representing highly and consistently transcribed *Borrelia* genes such as 16S rRNA, could improve the sensitivity of detection of *Borrelia* spp. Backstedt et al. (*17*) reported that leptospiral RNA–based quantitative PCRs (qPCRs) for human blood were >100-fold more sensitive than a DNA-based approach. Furthermore, detection of 16S rRNA (e.g., by nucleic acid sequence–based amplification) can distinguish viable from nonviable bacterial cells (*18*).

Hematogenous dissemination is a major pathogenetic event in Lyme borreliosis. Investigations of level and duration of spirochetemia caused by *B. miyamotoi* are needed for understanding the pathogenesis of the infection, as well as development of new diagnostic tools. 

Our study had 3 goals. First, we sought to determine whether *B. miyamotoi* disease can be detected in its early stage by using PCR for borrelial 16S rRNA. Second, we assessed which molecule (DNA or RNA) is the best choice for this test. Third, we investigated the dynamics of *B. miyamotoi* spirochetemia during the acute phase of the disease.

## Methods

### Study Design

Patients suspected of having tickborne diseases in Russia are hospitalized if they have been bitten by a tick and show development of signs and symptoms of acute infection (fever, chills, headache, fatigue, muscle aches) within a few weeks. A diagnosis of acute Lyme borreliosis was determined by the presence of erythema migrans, detection of borrelial IgM and IgG seroconversion, and detection of pathogen DNA or RNA. In this study, the case definition for *B. miyamotoi* disease was based only on PCR results. We have previously reported detailed information about case definitions for *B. miyamotoi* disease (*3*).

A total of 459 patients admitted to Municipal Clinical Hospital No. 33 (Medical Association Novaya Bolnitsa) in Yekaterinburg, Russia, during May–August 2009 and June–July 2010 for suspected tickborne infection were enrolled in this study. We obtained blood samples from all patients during the first 3 days of hospitalization for analysis by qPCR and ELISA. We performed PCR for detection of 16S rRNA of *B. miyamotoi*, *B. burgdorferi* sensu lato, *Ehrlichia chaffeensis*, and *E. muris*; the 5′-untranslated region gene of tickborne encephalitis virus (TBEV); and the major outer membrane protein 2 gene of *Anaplasma phagocytophillum* as described (*3*). 

We tested serum samples for borrelial IgM and IgG and TBEV IgM by ELISA (EUROIMMUN AG, Lubeck, Germany). We estimated the number of copies of *B. miyamotoi* and *B. burgdorferi* sensu lato DNA and RNA in PCR-positive patient blood samples by using a qPCR for 16S rRNA.

For determining duration of spirochetemia, we collected repeat blood samples from 9 PCR-positive *B. miyamotoi* disease patients in 2010 and 14 *B. miyamotoi* disease patients admitted to Municipal Clinical Hospital No. 33 in June–July 2015. We collected blood samples from these patients during the first 3 days after admission and then repeatedly for 4 days after initial detection of *B. miyamotoi* in the blood of these patients.

### Preparation of Blood Samples

We used differential centrifugation to separate spirochetes from erythrocytes. Blood samples were centrifuged at 160 × *g* for 10 min to pellet erythrocytes. We then transferred 500 μL of leukocytes and platelet-rich plasma into 1.5-mL tubes for centrifugation at 10,000 × *g* for 10 min to collect leukocytes and spirochetes. We extracted DNA and RNA from the pellet and 100 μL of the supernatant by using an AmpliSens Riboprep Kit (Central Research Institute of Epidemiology, Moscow, Russia). After cell lysis, we divided samples into 2 equal aliquots for separate isolation of *B. miyamotoi* RNA and DNA targets of 16S rRNA. We extracted RNA by using a universal internal RNA recombinant control having a known number of RNA copies per milliliter and DNA by using a universal internal DNA recombinant control having a known number of DNA copies per milliliter. RNA samples were not treated with DNase; therefore, RNA concentrations in this article are RNA/DNA concentrations.

### Molecular Detection of Infection

We performed PCR-based detection for TBEV, *B. burgdorferi* sensu lato, *A. phagocytophillum*, *E. chaffeensis*, and *E. muris* by using a commercial multiplex PCR kit (AmpliSens TBEV, *B. burgdorferi* sensu lato, *A. phagocytophillum*, *E. chaffeensis*/*E. muris*-FL; Central Research Institute of Epidemiology) according to the manufacturer’s instructions. We used the *B. miyamotoi*–specific primers Brm1 5′-CGCTGTAAACGATGCACACTTGGTGTTAATC-3′ (forward) and Brm2 5′-CGGCAGTCTCGTCTGAGTCCCCATCT-3′ (reverse) at concentrations of 360 nmol/L. The corresponding dye-labeled probe (final concentration 100 nmol/L) used was Brm-R6G-5′-CCTGGGGAGTATGTTCGCAAGAATGAAACTC-3′-BQH1. PCR conditions were 50°C for 15 min; 95°C for 15 min; 10 cycles at 95°C for 20 s, 67°C for 50 s, and 72°C for 20 s; and 40 cycles at 95°C for 20 s, 60°C for 50 s, and 72°C for 20 s. The fluorescence signal was recorded at the 60°C step for the last 40 cycles. Each run included negative controls and 2 positive recombinant DNA controls (a *B. miyamotoi* 16S rRNA gene fragment and an internal control having 10^4^–10^6^ copies/mL) as standards. 

We used *B. burgdorferi* sensu lato–specific primers Brb1 5′-TGCAAGTCAAACGGGATGTAGCAATACA-3′ (forward) and Brb2 5′-GGCTTCCTTTCATCAATTAACAAA-3′ (reverse) at concentrations of 360 nmol/L. The corresponding dye-labeled probe (final concentration 100 nmol/L) was Brb-R6G-5′-TAGGTAGAT-BQH1-CATCCACGCGTTACTACC-3′. PCR conditions were 50°C for 15 min; 95°C for 15 min; and 45 cycles at 95°C for 10 s and 60°C for 20 s. The fluorescence signal was recorded at the 60°C step for the last 45 cycles. Each run included negative controls and 2 positive recombinant DNA controls (a *B. burgdorferi* sensu lato 16S rRNA gene fragment and an internal control having 10^4^–10^6^ copies/mL) as standards.

The RNA template was subjected to reverse transcription (Thermo Fisher Scientific, Waltham, MA, USA). In 2015, a commercial PCR kit (AmpliSens *B.miyamotoi*-FL; Central Research Institute of Epidemiology) was used according to the manufacturer’s instructions for *B. miyamotoi* *glpQ* gene screening of patients with suspected tickborne diseases.

### ELISA

We tested serum samples collected at the time of hospitalization and 1–2 weeks later for borrelial IgM and IgG. We obtained serologic evidence of exposure to *Borrelia* spp. by using 2 ELISAs (EI 2132–9601 M and EI 2132–9601–2 G; EUROIMMUN AG) and detected TBEV IgM by using a semiquantitative ELISA (EI 2661–9601 M; EUROIMMUN AG).

### Data Analysis

We used the Wilcoxon signed-rank test for comparing copy numbers of *B. miyamotoi* RNA and DNA in blood samples and the Kaplan–Meier estimator to assess persistence of DNA or RNA of pathogens in blood. We performed time-to-event analysis by using the following assumptions: 1) starting time was the first day of illness; 2) observations for no antimicrobial drug treatment were complete data; 3) observations for late antimicrobial drug treatment (when DNA or RNA were eliminated from blood before start of treatment) were complete data; and 4) observations for antimicrobial drug treatment were incomplete data (right censored). The Kaplan–Meier estimator assumes that at any time, patients who are censored have the same survival prospects as those who continue to be followed up (*19*).

We used the Clopper–Pearson interval for calculating CIs for proportions. Descriptive statistics are given as mean and SD or median and interquartile range (IQR). We analyzed data by using SPSS software (IBM, Armonk, NY, USA) or R software (https://www.r-project.org/).

## Results

### Study Population

During 2009–2010, of 310 (67.5%, 95% CI 63.0%–71.8%) patients with borreliosis, 34 (7.4%, 95% CI 5.2%–10.2%) patients were positive for TBEV, and 115 (25.1%, 95% CI 21.2%–29.3%) were positive for other inflammatory diseases. Genetic markers of anaplasmosis and ehrlichiosis were not detected.

Erythema migrans as a symptom was observed in 167 (53.9%, 95% CI 48.1%–59.5%) patients with borreliosis and was absent in the remaining 143 (46.1%, 95% CI 40.5%–51.9%) patients. Among patients with erythema migrans, DNA or RNA of the *B. burgdorferi* sensu lato 16S rRNA gene was detected by qPCR in 18.6% (95% CI 13.0%–25.3%), and 3.0% (95% CI 0.4%–5.2%) were positive for *B. miyamotoi* (Table 1). For patients without erythema migrans, genetic markers of *B. burgdorferi* sensu lato were found in 3.5% (95% CI 1.1%–8.0%), and 45.5% (95% CI 37.1%–54.0%) were positive for *B. miyamotoi*. One case-patient was co-infected with *B. burgdorferi* sensu lato and *B. miyamotoi*. For 51.0% (95% CI 42.6%–59.5%) of patients, borreliosis was confirmed by determination of antibody seroconversion by ELISA only.

**Table 1:** Cross-validation of detection of *Borrelia* spp. by quantitative PCR and ELISA in blood samples from 310 patients with suspected borreliosis, Yekaterinburg, Russia, 2010 and 2015*

Quantitative PCR for 16S rRNA gene	No. patients	No. ELISA positive	No. ELISA negative	ND
With erythema migrans				
* Borrelia burgdorferi* sensu lato	31	30	1	0
* B. miyamotoi*	5	5	0	0
PCR negative	131	81	39	11
Total	167	116	40	11
Without erythema migrans				
* B. burgdorferi* sensu lato	4	4	0	0
* B. miyamotoi*	65	55	10	0
* B. burgdorferi* sensu lato plus *B. miyamotoi*	1	0	1	0
PCR negative	73	73	0	0
Total	143	132	11	0

*ELISA results are for borrelial IgM or IgG seroconversion. ND, not determined.

### RNA and DNA Concentrations

Because onset of *B. miyamotoi* disease is acute, patients are usually admitted to a hospital during the first 3 days of the disease in Russia. In our study, 79% of patients were admitted to the hospital during the first 3 days, which indicates that concentrations of *B. miyamotoi* RNA or DNA are accurate for this period.

**Figure 1:**
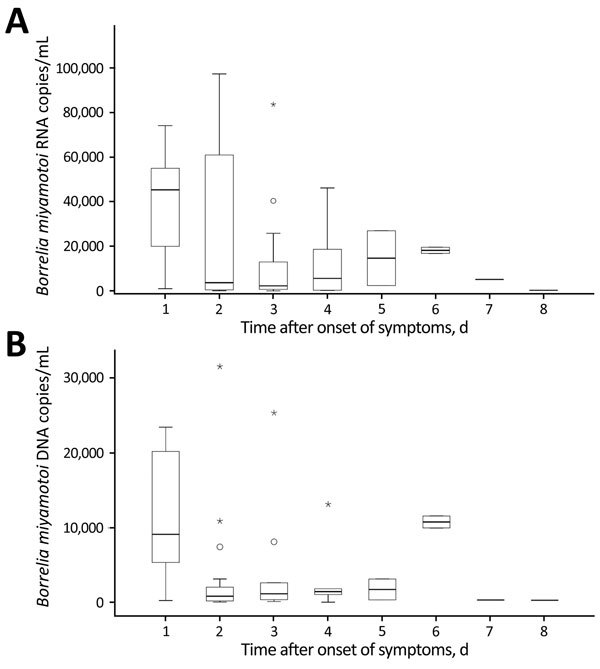
Concentration of *Borrelia miyamotoi* A) RNA and B) DNA in blood samples from patients with *B. miyamotoi* disease during disease progression, Yekaterinburg, Russia, 2009–2010. Blood samples were obtained before antimicrobial drug therapy was given. Boxes indicate interquartile ranges (IQRs), horizontal bars indicate medians, and error bars indicate 1.5× IQR. Circles indicate moderate outliers (1.5×–3× IQR, 238,700 copies/mL), and asterisks indicate extreme outliers (>3× IQR, 280,000–420,000 copies/mL).

The maximum pathogen RNA concentration was observed on day 1 of the disease (Figure 1, panel A). On day 2, RNA copy number varied widely (median 3,700–45,360 copies/mL); it remained in this range on subsequent days. The concentration of *B. miyamotoi* DNA varied in a similar way (Figure 1, panel B); the highest value of 9,085 copies/mL was found on the first day, after which the value decreased to 797.5 copies/mL. Because there were only 2 observations on day 6, observed ranges of DNA (and RNA) concentrations are speculative. The Wilcoxon signed-rank test showed that the copy number of RNA in blood was significantly higher than the copy number of DNA (p<0.001). The RNA:DNA ratio also showed a wide range (median ratio 3.8, IQR 2.1–7.5).

Early Lyme disease is usually manifested only by erythema migrans; thus, patients are often hospitalized late in their illness. The median time gap between onset of disease and hospitalization was 6 days (IQR 3–9 days). We showed that PCR diagnosis of Lyme disease has low sensitivity. Consequently, *B. burgdorferi* sensu lato RNA and DNA concentration varied; the median RNA concentration was 585.0 copies/mL (IQR 305.3–1,392.5 copies/mL), and the median DNA concentration was 19.9 copies/mL (IQR 8.1–121.2 copies/mL). The concentration of *B. burgdorferi* sensu lato RNA was also significantly higher than that for DNA (p<0.001), which resulted in a greater RNA:DNA ratio (median 40.9, IQR 13.3–77.4). Differences between *B. miyamotoi* and *B. burgdorferi* sensu lato for concentrations of RNA and DNA were significant (z = –5.77, p<0.001, by Mann-Whitney U-test).

**Figure 2:**
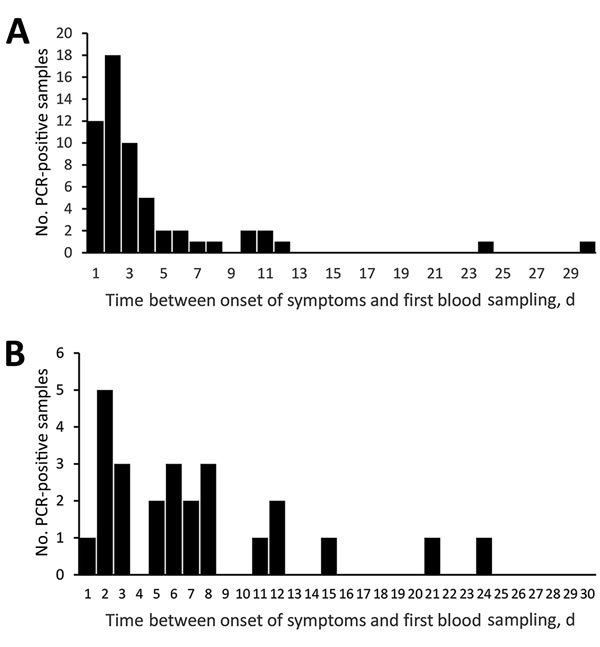
Distribution of PCR positive blood samples obtained after onset of symptoms for patients infected with A) *Borrelia miyamotoi* or B) *B. burgdorferi* sensu lato, Yekaterinburg, Russia, 2009–2010. Blood samples were obtained before antimicrobial drug therapy was given.

*B. miyamotoi* DNA or RNA were detected in blood samples up to day 30 of disease (Figure 2, panel A). However, despite such a wide range, 90% of all observations were in the first 8 days of the disease. We showed in a previous study (*20*) that 7 (9.1%) positive samples, which were obtained from patients given a diagnosis of *B. miyamotoi* disease during the second week of disease (or later), were assumed to be caused by a relapse of fever. However, this assumption could not be confirmed because of lack of availability of clinical materials. Pathogen DNA or RNA were detected in blood samples from patients with Lyme borreliosis obtained up to day 24 of disease (Figure 2, panel B).

The study showed a high number of false-negative PCR results in patients with and without EM (Table 1). The time gap between onset of disease and blood sampling can affect results of blood tests. Therefore, all blood samples from the group of patients without EM were obtained based on the time after onset of symptoms. We found that blood samples from *B. miyamotoi* PCR-positive patients were obtained significantly earlier (z = –3.29, p<0.001, by Mann-Whitney U-test). The median time of blood sampling was 2.0 days (IQR 2.0–4.0 days) for PCR-positive patients without EM and 4.0 days (IQR 2.3–6.0 days) for PCR-negative patients without EM after initial symptoms. Therefore, PCR is applicable for diagnosis of *B. miyamotoi* disease only during the first few days of the disease.

### Duration of Spirochetemia

**Figure 3:**
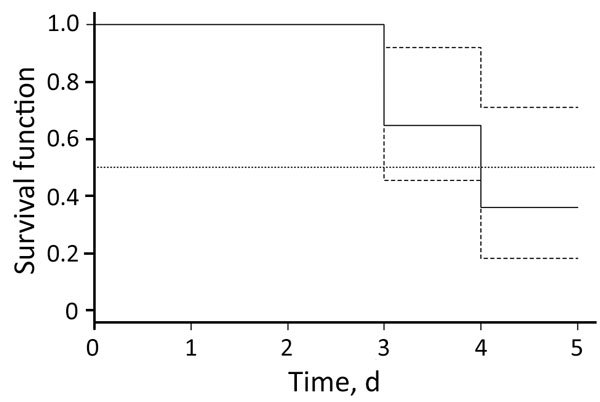
Kaplan–Meier estimates of *Borrelia miyamotoi* RNA or DNA in blood samples from patients with *B. miyamotoi* disease (solid line), Yekaterinburg, Russia, 2009–2010. Dashed line indicates 95% CIs, and dotted line indicates median. Observations during antimicrobial drug therapy represent incomplete data (right censored).

In 2010 and 2015, we obtained blood samples repeatedly during the first 6 days of disease from 23 patients with confirmed *B. miyamotoi* disease (Table 2) and used data for these samples to estimate the duration of spirochetemia. We excluded samples from patients who had started antimicrobial drug therapy before or at the same time as the blood tests and those from patients who had a time gap between blood samples **>**2 days. We performed time-to-event analysis for 17 patients. Pathogen DNA or RNA was detected in blood up to day 5 of illness (Table 2), and all samples showed negative results on day 6 of illness. Median time for detection of pathogen DNA or RNA in blood was 4.0 days (95% CI 3.1–4.9 days) (Figure 3).

**Table 2:** Dynamics of *Borrelia miyamotoi* RNA and DNA load in blood samples from 23 patients with *Borrelia miyamotoi* disease, Yekaterinburg, Russia, 2010 and 2015*

Year and patient ID	PCR product for *B. miyamotoi*, copies/mL									
	Day 1		Day 2		Day 3		Day 4		Day 5	
	RNA	DNA	RNA	DNA	RNA	DNA	RNA	DNA	RNA	DNA
2010										
1	NA	NA	1.9 × 10^4^	3.3 × 10^4^	–	–	NA	NA	NA	NA
2	NA	NA	1.9 × 10^4^	5.5 × 10^4^	**NA**	**NA**	NA	NA	3.1 × 10^3^	2.7 × 10^4^
3	NA	NA	2.0 × 10^3^	2.4 × 10^3^	–	–	**–**	**–**	–	–
4	NA	NA	9.5 × 10^2^	3.4 × 10^3^	3.4 × 10^2^	7.1 × 10^2^	–	–	–	–
5	NA	NA	1.6 × 10^3^	1.2 × 10^4^	**–**	**8.5 × 10^1^**	–	–	–	–
6	NA	NA	NA	NA	1.1 × 10^3^	2.6 × 10^4^	**–**	**–**	NA	NA
7	NA	NA	6.7 × 10^3^	2.7 × 10^4^	–	–	**NA**	**NA**	NA	NA
8	NA	NA	6.5 × 10^2^	2.8 × 10^3^	–	–	**NA**	**NA**	NA	NA
9	NA	NA	9.9 × 10^3^	1.7 × 10^4^	–	–	NA	NA	NA	NA
2015										
10	NA	NA	1.2 × 10^4^	8.2 × 10^3^	**2.2 × 10^1^**	**–**	–	–	–	–
11	NA	NA	**2.8 × 10^5^**	**9.2 × 10^4^**	**1.0 × 10^3^**	**3.2 × 10^2^**	–	–	–	–
12	NA	NA	5.4 × 10^4^	1.7 × 10^4^	**–**	**–**	–	–	–	–
13	NA	NA	**8.7 × 10^4^**	**8.8 × 10^3^**	–	–	–	–	–	–
14	NA	NA	6.1 × 10^4^	3.2 × 10^4^	**NA**	**NA**	–	–	–	–
15	NA	NA	NA	NA	1.8 × 10^3^	1.3 × 10^3^	**NA**	**NA**	NA	NA
16	**2.0 × 10^4^**	**1.9 × 10^4^**	NA	NA	NA	NA	2.0 × 10^2^	1.3 × 10^1^	NA	NA
17	NA	NA	NA	NA	3.4 × 10^3^	6.9 × 10^2^	–	–	–	–
18	NA	NA	**1.9 × 10^4^**	**7.2 × 10^4^**	–	–	–	–	–	–
19	NA	NA	4.3 × 10^4^	1.4 × 10^4^	9.7 × 10^1^	3.0 × 10^1^	–	–	–	–
20	NA	NA	4.2 × 10^5^	9.6 × 10^4^	–	–	3.2 × 10^2^	8.0 × 10^1^	**–**	**–**
21	NA	NA	2.9 × 10^2^	6.5 × 10^1^	–	–	**–**	**–**	–	–
22	NA	NA	NA	NA	3.3 × 10^2^	2.3 × 10^3^	–	–	–	–
23	NA	NA	**NA**	**NA**	1.3 × 10^4^	4.1 × 10^3^	NA	NA	–	–

*Day indicates day after onset of symptoms. Bold indicates day of starting antimicrobial drug therapy (ceftriaxone). For patients 1, 4, 9, 17, 19, and 22, antimicrobial drug therapy was started on day 6 or later. ID, patient identification; NA, not available (blood sample was not obtained); –, negative.

## Discussion

Borreliosis is the most prevalent tickborne disease in Russia (*21**,**22*). Our findings showed that, in Yekaterinburg, ≈23% cases of borreliosis (confirmed clinically, serologically, or by PCR) were caused *B. miyamotoi*. Findings that indicated that *B. miyamotoi* disease might not be a rare infection have been previously suggested for the United States (*8*,*14*,*23*) and the Netherlands (*13*).

We found that erythema migrans was not apparent in 70 (93%) patients with *B. miyamotoi* disease, and only in 7% of case-patients was erythema migrans manifested. We obtained data for the 5 PCR-confirmed case-patients with *B. miyamotoi* disease and erythema migrans and the case-patient co-infected with *B. burgdorferi* sensu lato and *B. miyamotoi*. We believe that these 5 patients were also co-infected with *Borrelia* spp. and that the *B. burgdorferi* sensu lato DNA was not detected because of the low sensitivity of the PCR. Additional research is needed to test this assumption. Recent studies reported that in the United States, 14% of patients with *B. miyamotoi* disease were co-infected with *B. burgdorferi* (*7*,*23*), including a patient with erythema migrans (*23*).

 Our study showed poor sensitivity (≈11%) of qPCR detection for 16S rRNA for *B. burgdorferi* sensu lato. In comparison, the sensitivity of PCR for *B. burgdorferi* sensu lato varied widely (7.5%–78.1%) (*12*,*24*,*25*). Several studies have reported low sensitivity of PCR for *B. burgdorferi* sensu lato DNA for routine diagnostic purposes because of low numbers of spirochetes circulating in the bloodstream during acute infection (*8*,*12*,*26**–**29*). For relapsing fever caused by *Borrelia* spp., the number of *glpQ* gene copies for *B. duttonii and B. reccurentis* ranged from 10^2^ to 10^5^ copies/mL for 7 (0.3%) of 2,057 healthy participants and from 10^3^ to 10^8^ copies/mL for 15 (3.9%) of 382 patients with fever who were surveyed in Tanzania (*16*). The number of copies of the *B. miyamotoi* gene in serum samples of 2 patients in Japan was 7.2 × 10^3^ and 2.8 × 10^4^ copies/mL by 16S rRNA qPCR (*30*). A mean copy number of 7,787 copies/mL was reported for *B. miyamotoi* disease patients in the northeastern United States (*23*).

The number of copies of the *B. miyamotoi* gene in serum samples from 2 patients in Japan was 7.2 × 10^3^ and 2.8 × 10^4^ copies/mL by 16S rRNA qPCR (*30*). A mean copy number of 7,787 copies/mL was reported for *B. miyamotoi* disease patients in the northeastern United States (*23*). Our study identified the bacterial load in blood samples of patients with *B. miyamotoi* disease (<9,085 copies/mL) and showed that an early diagnosis of this disease is possible if a PCR for the 16S rRNA gene is used. The concentration of *B. miyamotoi* RNA in blood is higher than that for DNA. We found a low median RNA:DNA ratio (≈4), which indicates that use of RNA as the target molecule is inappropriate.

We obtained data on the duration of spirochetemia, which contributes to the early diagnosis of *B. miyamotoi* disease. It is possible to detect borrelial DNA by PCR during the first 3 days of the disease. However, bacterial DNA is then no longer detectable in the blood, so PCR detection is ineffective after 4 days. We detected 73 *Borrelia* spp. ELISA-positive patients without erythema migrans who seroconverted within the observation period and were PCR negative for both *Borrelia* spp. On average, these samples were obtained later than *B. miyamotoi* PCR-positive samples, suggesting that they might have been collected after the end of the period of spirochetemia and caused the PCR-negative results.

We characterized the duration of *B. miyamotoi* spirochetemia during acute illness. Although there was no strong clinical confirmation, our data showed that *B. miyamotoi* DNA and RNA might be detected in the circulation within 30 days after onset of disease. This extended parasitemia is probably related to disease relapse (*20*). Lee et al. (*8*) provided indirect results for *B. miyamotoi* spirochetemia and detected *B. miyamotoi* DNA in blood samples of 4 patients during the period with little tick exposure in the northeastern United States. These authors suggested that this off-season spirochetemia with a low bacterial density was most likely the result of bacteria being dislodged periodically from persistent deep-tissue lesions (*8*). Thus, a long-term study of pathogen persistence is required.

The duration of *B. miyamotoi* spirochetemia is relatively short. Thus, the true number of ambulatory and hospitalized patients infected with *B. miyamotoi* will not be known until a sensitive, reliable, diagnostic laboratory test (i.e., serologic test) is available to detect causative agents in patients with acute infections in disease-endemic areas.
